# Massage Therapy to Control Anxiety Before Extraction of an Impacted Tooth

**DOI:** 10.7759/cureus.29893

**Published:** 2022-10-04

**Authors:** Ramesh Kunusoth, Shreya Colvenkar, Aditya Mohan Alwala, Shalini Sampreethi, MD Shakeel Ahmed

**Affiliations:** 1 Department of Oral and Maxillofacial Surgery, MNR Dental College and Hospital, Sangareddy, IND; 2 Department of Prosthodontics, MNR Dental College and Hospital, Sangareddy, IND

**Keywords:** injection, anesthesia, massage, extraction, anxiety

## Abstract

Anxiety creates a stressful situation for the dentist as well as the patient. This article describes a simple technique to manage anxiety using an eye massager. The audio and visual distraction technique helps the patient relax and complete the procedure. This article also stresses some basic skills like good communication skills and establishing mutually trustworthy dentist-patient relationships to enhance patient satisfaction during treatment. Managing anxiety during dental visits will help improve oral health in the long term.

## Introduction

Many patients experience anxiety during their dental visits. This fear can be mild or moderate, but when this fear becomes severe, it creates a stressful experience for both the dentist as well as the patient.

Anxiety could be the result of a direct negative dental experience [1], fear of needles during anesthesia [2], and fear of gagging or choking [3]. The literature search also mentions that people who experience dental anxiety are more likely to have several other comorbid psychological conditions [4].

Out of fear, patients are much more likely to cancel or fail to show up for their regular dental appointments. Such patients will have poor oral health, requiring complex treatment [5,6]. Even if such patients show up for their appointment, dentists may find them difficult to treat and need to spend more time on treatment.

Various treatment options are mentioned in the literature to manage anxiety, depending on the severity. They can be categorized into pharmacological and non-pharmacological means [3,6-13]. However, only the dentist will be better able to judge which treatment will be appropriate depending on the level of anxiety.

This case report describes a simple technique to manage anxious patients before tooth extraction.

## Case presentation

**Figure 1:**
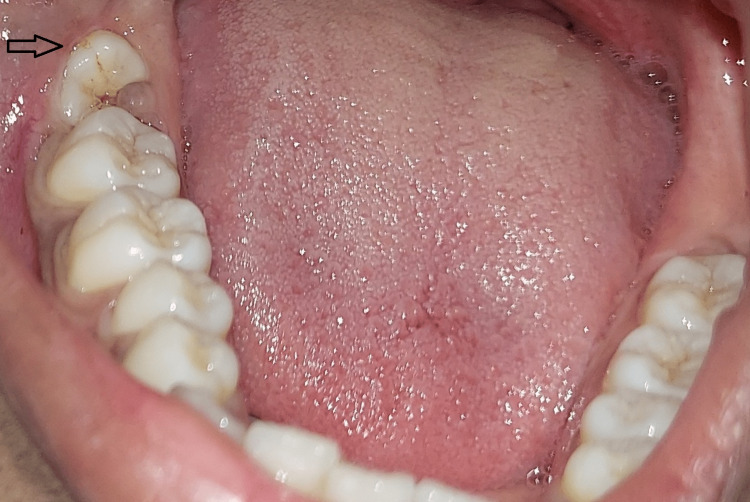
Impacted third molar

An 18-year-old female patient visited the department of oral and maxillofacial surgery for extraction of an impacted mandibular third molar (Figure 1). The patient experienced pain and food impaction in relation to the impacted tooth.

The patient revealed she was very anxious about tooth extraction, as she had a negative experience during childhood. As a child, she had a phobia of needle injections during childhood. The dentist she was seeing then had raised his voice and forcefully injected anesthesia by holding her hands. This understandably left her psychologically scared and fearful of any dental procedures. This appointment was only to build a good rapport and build trust with the patient. The patient was calmed down and relaxed by genuinely acknowledging her concerns. Once the patient was relaxed, the procedure that was planned to be carried out in her next appointment was explained to her.

The patient was asked to fill out the Modified Dental Anxiety Scale (MDAS) questionnaire consisting of five questions:

1. If you went to your dentist for treatment tomorrow, how would you feel?

2. If you were sitting in the waiting room (waiting for treatment), how would you feel?

3. If you were about to have a tooth drilled, how would you feel?

4. If you were about to have your teeth scaled and polished, how would you feel?

5. If you were about to have a local anesthetic injection in your gum, above an upper back tooth, how would you feel?

It has a consistent answering scheme for each question, ranging from not anxious, slightly anxious, fairly anxious, very anxious, and extremely anxious. Each response is scored from 1 to 5. So, the not anxious response is scored 1, slightly anxious is scored 2, fairly anxious is scored 3, very anxious is scored 4, and extremely anxious response is scored 5. The total score of this scale ranges from 5 to 25 with cut-off scores of 14 and 19 suggestive of high dental anxiety and dental phobia, respectively. The patient's pulse, heart rate, and blood pressure were also monitored. The patient was prescribed pain relief medication and antibiotics and asked to visit after three days.

Second visit

The patient was given the MDAS questionnaire to complete. The patient's vitals were monitored, and she was provided information about the procedure to answer any misconceptions she may have about the treatment. Both sensory (slight needle prick, pressure, numbness), as well as procedural, information was explained to the patient. The word injection in itself raised the patient's anxieties. The patient was explained that it was a mild prick, and it was needed for anesthesia to take effect so that the procedure would be pain-free afterward. Here systemic desensitization was used on the patient The patient was shown the syringe, explained its parts, and was asked to hold the syringe till she didn’t feel any anxiety. Later, the syringe was placed in her mouth with the cap on. But when the syringe without the cap was inserted, the patient did not allow it.

**Figure 2:**
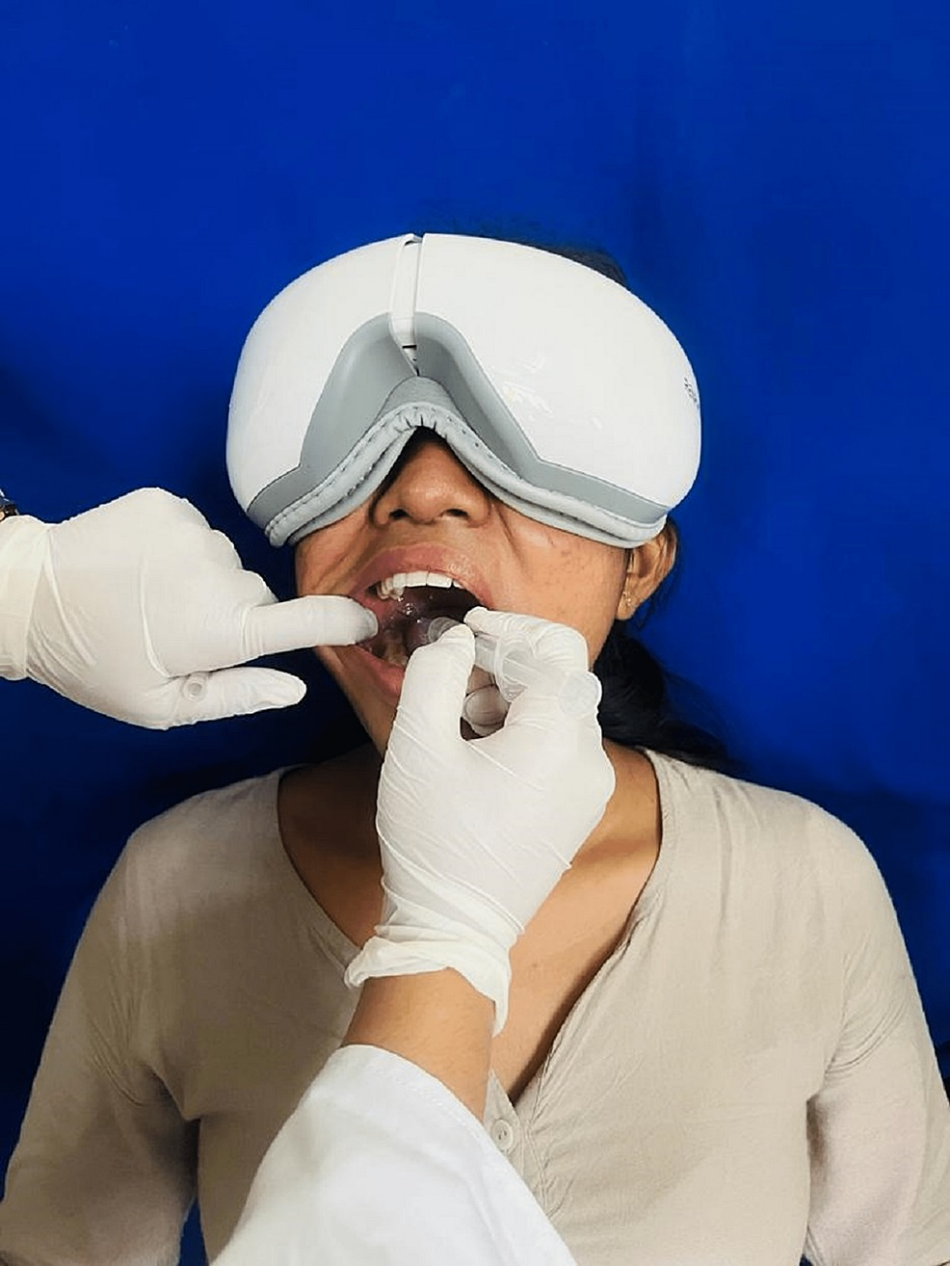
Patient with eye massager

The patient was given a break and asked to perform relaxation breathing. The patient said she was visually scared of that syringe. So, a simple technique of using an eye massager to relax, as well as block the patient's view, was tried after obtaining her consent. She was asked to wear an eye massager (Figure 2).

**Figure 3:**
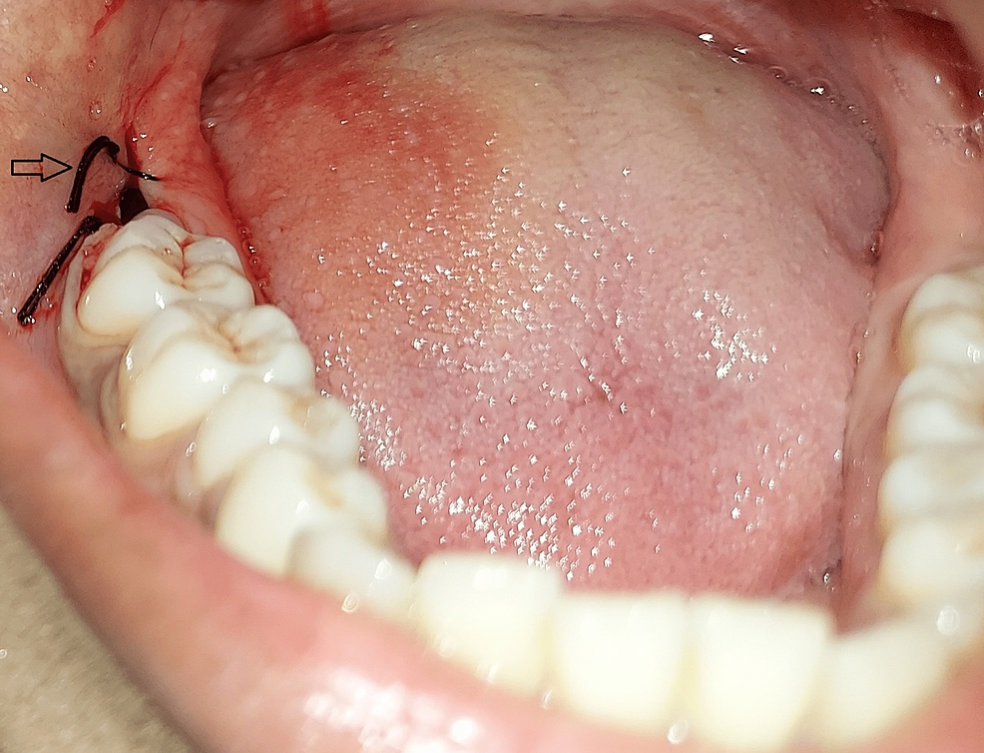
Mandibular third molar extracted and sutured

She was also asked to signal by raising her hand to stop the procedure if needed. After 10 minutes of relaxation, she was asked to open her mouth. Topical anesthesia gel was applied, and local anesthesia was injected. The patient did feel the prick but not to an extent that she could not tolerate it. Once anesthesia was achieved, the patient was asked to remove the eye massager. Tooth extraction was carried out, and the extraction site was sutured (Figure 3).

The patient was given post-extraction instructions and recalled after five days. The patient's vitals were again monitored, and she was asked to fill out the anxiety questionnaire again.

Third visit

The patient was very relaxed during this appointment. The extraction site showed good signs of healing. She was asked to again fill out the MDAS questionnaire.

## Discussion

Dental anxiety can be caused due to various factors. It is very important to identify its etiology to adopt a proper treatment approach tailored to the patient’s concerns [7]. Before starting the treatment, it is crucial to establish a good rapport and communication with the patient [8].

The patient revealed a negative experience during her childhood. The raised tone of voice used by the dentist and the forceful holding of her hands during her childhood created lasting distrust in the child. So, caution is necessary when using this technique, as it can do more harm than good [9].

In the present case, the patient was received with a calm and friendly attitude. Proper history was taken to understand the cause of fear, and realistic information about dental treatment was provided. This helped in building a trusting relationship with the patient. Hamasaki and colleagues reported that patients who felt positive communication with their dentists had better satisfaction and less fear than those with less communication with their dentists [8].

A rest break was given to the patient when needed. Rest breaks enhance the patient’s sense of equivalency and control over the dental procedure. It also allows the patient to calm down prior to becoming anxious and continue the treatment [10]. The patient was asked to perform relaxation breathing during breaks. Park E and his colleagues found that relaxation breathing does appear to lower anxiety as well as perceived pain [11].

Systematic desensitization was used by gradually exposing a fearful stimulus until the patient felt little or no anxiety. It is shown to be effective in reducing dental fear [12]. But this technique did not help in this situation.

The patient's anxiety score recorded with the help of the Modified Anxiety Scale depicted extremely anxious on the first visit. Pulse, heart rate, and blood pressure were slightly high before the procedure. After the procedure, the patient's highly anxious score was drastically reduced to not anxious. Blood pressure, pulse, and heart rate were also within the normal range. When the patient visited on the third day, she was willing to do regular checkups.

Audiovisual distraction is found to be a powerful tool to manage anxious patients [3,13]. The patient feared injection so a visual and auditory distraction technique using an eye massager was tried. The eye massager relaxed the patient together with blocking the site from fearful stimuli. The dentist could patiently complete the procedure. Future research would concentrate on more patients to compare the effectiveness of massage therapy with other anxiety control methods.

## Conclusions

Dental anxiety creates a stressful situation for the patient as well as the dentist. This article describes a simple technique to manage anxiety using an eye massager. The audio and visual distraction using the eye massager helped the patient relax and complete the procedure.
